# Genetic analysis of mixed models of fruit sugar–acid fractions in a cross between jujube (*Ziziphus jujuba* Mill.) and wild jujube (*Z. acido jujuba*)

**DOI:** 10.3389/fpls.2023.1181903

**Published:** 2023-05-12

**Authors:** Yang Zhi, Zhang Chuanjiang, Yang Xinfang, Dong Mengyi, Wang Zhenlei, Yan Fenfen, Wu Cuiyun, Wang Jiurui, Liu Mengjun, Lin Minjuan

**Affiliations:** ^1^ The National and Local Joint Engineering Laboratory of High Efficiency and High Quality Cultivation and Deep Processing Technology of Characteristic Fruit Trees in Southern Xinjiang, the Production Engineering Laboratory of Characteristic Fruit Trees in Southern Xinjiang of Xinjiang Production and Construction Corps, College of Plant Science of Tarim University, Alar, Xinjiang, China; ^2^ Key Laboratory of Tarim Basin Biological Resources Protection and Utilization, Xinjiang Production and Construction Corps, Alar, Xinjiang, China; ^3^ College of Horticulture, Hebei Agricultural University, Baoding, Hebei, China; ^4^ Center of Chinese Jujube, Hebei Agricultural University, Baoding, Hebei, China

**Keywords:** jujuba, wild jujuba, hybridization, fruit, genetic predisposition, quantitative characters, major gene +polygene

## Abstract

Chinese jujube (*Ziziphus jujuba* Mill.), an economically significant species in the Rhamnaceae family, is a popular fruit tree in Asia. The sugar and acid concentrations in jujube are considerably higher than those in other plants. Due to the low kernel rate, it is extremely difficult to establish hybrid populations. Little is known about jujube evolution and domestication, particularly with regard to the role of the sugar and acid components of jujube. Therefore, we used cover net control as a hybridization technique for the cross-breeding of *Ziziphus jujuba* Mill and ‘JMS2’ and (*Z. acido jujuba*) ‘Xing16’ to obtain an F_1_ population (179 hybrid progeny). The sugar and acid levels in the F_1_ and parent fruit were determined by HPLC. The coefficient of variation ranged from 28.4 to 93.9%. The sucrose and quinic acid levels in the progeny were higher than those in the parents. The population showed continuous distributions with transgressive segregation on both sides. Analysis by the mixed major gene and polygene inheritance model was performed. It was found that glucose is controlled by one additive-dominant major gene and polygenes, malic acid is controlled by two additive-dominant major genes and polygenes, and oxalic acid and quinic acid are controlled by two additive-dominant-epistatic major genes and polygenes. The results of this study provide insights into the genetic predisposition and molecular mechanisms underlying the role of sugar acids in jujube fruit.

## 1 Introduction

Chinese jujube (*Ziziphus jujuba* Mill.) is one of the most economically important fruit trees in China. Wild jujube *(Z. jujuba* Mill. var. spinosa) trees, which originally produced small, acidic fruit, evolved further to produce larger and sweeter fruits, and these varieties were domesticated (Mingxin et al., 2020). Fruit development and ripening are closely regulated at the genetic and epigenetic levels, so the genetics of the domesticated variety are likely altered (Huang et al., 2016; Song et al., 2019).

The sugars and acids present in fruits play an important role in determining fruit taste. The sugar and acid levels in jujube fruit are higher than those in other fruits (Liu et al., 2020). The gene families involved in sugar metabolism show greater expansion in the jujube genome than in the genomes of other Rosales fruits (Liu et al., 2014). As carbon sources and quality-related factors, soluble sugars in fruits, such as sucrose, fructose, and glucose, play a crucial role in fruit cultivation. Cultivated and wild jujubes exhibit highly contrasting profiles for sugar and organic acid dynamics during the fruit ripening process. Sucrose accumulation occurs gradually, and the organic acid accumulation performance differs between cultivated and wild jujube fruit (Huang et al., 2016; Song et al., 2019). The distribution of acid components in jujube fruit is highly balanced. There is a strong correlation between the sources and sinks of sugar in fruits. It has been shown that sugar transporter genes are highly expressed during the ripening of jujube fruit, which explains the high sugar accumulation in these fruit (Zhang et al., 2016). During jujube domestication, a series of genes involved in the key steps of sugar metabolism and organic acid metabolism underwent selection, and their transcript levels were enhanced in cultivated jujube compared to wild jujube (Huang et al., 2016; Song et al., 2019). There is also evidence that the sugar unloading systems differ between cultivated and wild jujube, as plasmodesmata are present in the cultivars but absent in wild jujube. It has been shown that sugar transporter genes are highly expressed during the ripening of jujube fruit, which explains their high sugar accumulation (Zhang et al., 2016).

Since Mendel’s study, research on the quantitative genetic basis of plant traits has been commonly conducted by using the frequency distribution of segregation representative types and genotypes (Porter and Theodore, 2014). In plant genetics, the quantitative trait genetic system is the gene system that affects plant traits in a quantitative manner. It is possible to determine how many plant traits are controlled by various genes and the genetic effects of individual genes by dividing them into major genes and minor polygenes, which coexist and affect plant phenotypes. (Gai et al., 2000). A method for separating and analyzing a mixed genetic model of major genes and polygenes has been developed. Gai Junyi regards the mode of mixed inheritance as the main gene + polygene genetic system (Gai et al., 2000), which represents a major polygene hybrid genetic model for identifying quantitative traits and estimating related genetic parameters. This method can be applied to the analysis of multiple or single genetic generations. A separation and analysis method for identifying quantitative traits has been proposed (Wang et al., 2001). In recent years, this method has been applied to food crops (Chen et al., 2009; Zhang et al., 2012), vegetables (Zhang et al., 2006; Qiang et al., 2014), fruits (Pastina et al., 2011; Fujikawa and Sawada, 2016), and flowers to analyze the genetic basis of segregation in plants.

Understanding the differences and genetics of wild and cultivated jujubes and their hybrid progeny in terms of the levels of fruit sugar–acid components will provide valuable information for the genetic improvement of jujube. Due to the extremely low fruit setting rate of jujube, it is difficult to cross jujube trees (Guo et al., 2019; Du et al., 2023), and there has been no genetic analysis reported for the sugar and acid components in jujube fruit under hybridization conditions. In this study, we established a cross-genetic population study of jujube and analyzed the genetic tendencies of different sugar and acid components. In addition, to study the inheritance of these components in jujube (Fenfen et al., 2020), we used hybrid genetic analysis to determine their genetic effects (Fernando et al., 1994).

## 2 Materials and methods

### 2.1 Plant material and growth conditions

**Figure 1:**
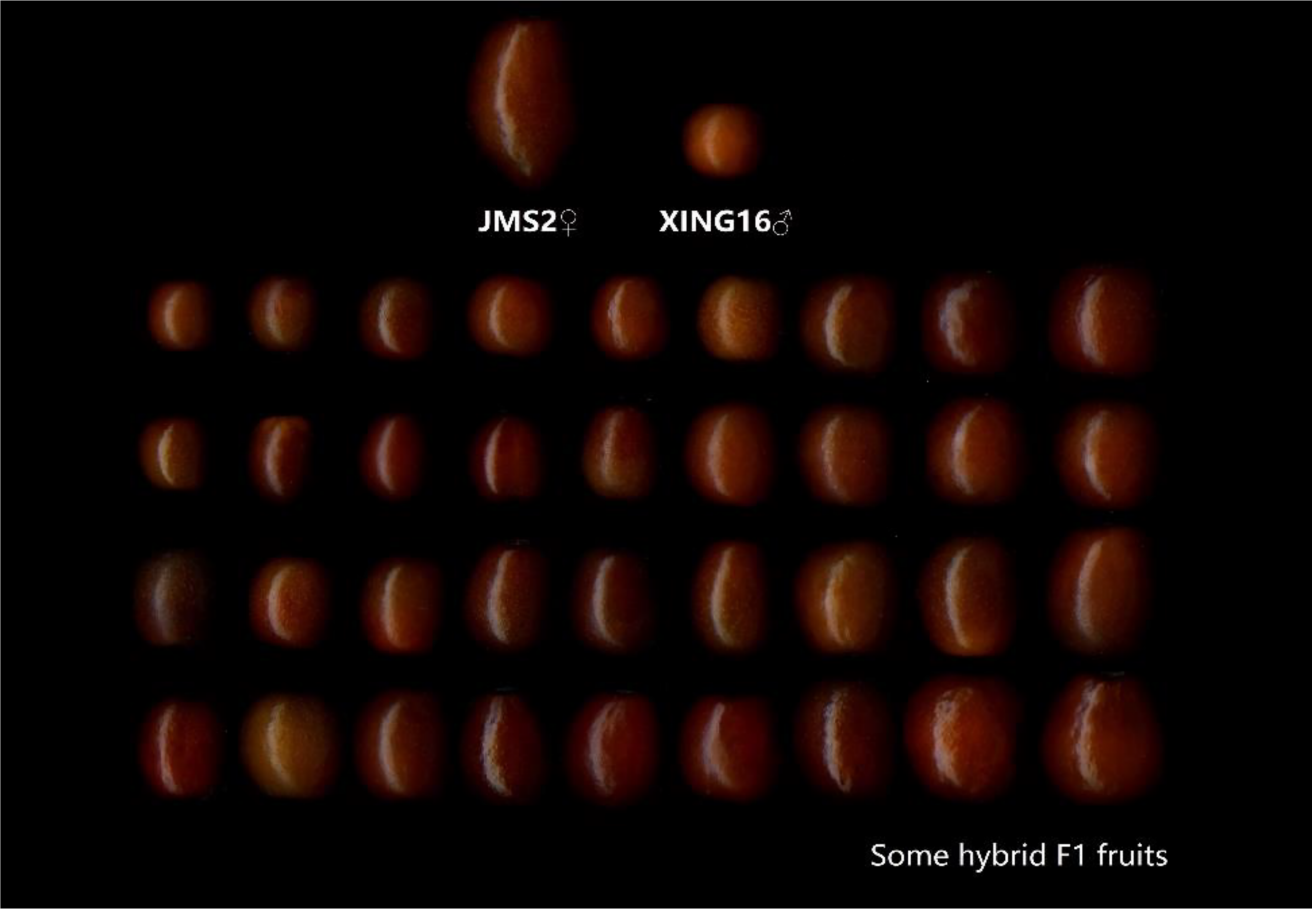
Fruit of parents and some F1 hybrids.

In 2015, in the Jujuba Breeding Base of Hebei Agricultural University in the 7th Company of the 10th Regiment of the First Division of Xinjiang Production and Construction Corps, the male sterility jujube variety ‘JMS2’ and wild jujube ‘Xing 16’ (as parents) were enclosed together in a netted room, and bees were used to help pollinate. In the same year, the fruit were harvested, the kernels were removed, and the descendants were obtained by sowing. A total of 179 true hybrid progeny were obtained, as shown through the SSR identification (Fenfen et al., 2018). The fruit of the parents and some hybrids are shown in **Figure 1**. The parents and their 179 hybrid progeny were used as test materials to investigate and analyze the sugar–acid-related indicators for two consecutive years (2019 and 2020). For each individual tree, 10 healthy and pest-free fruit were randomly selected from four directions, in the southeast, northwest, and periphery of the crown, and the materials were sent back to the laboratory on the day of collection for sample treatment and the determination of various indicators.

### 2.2 Determination of sugar content

Determination of the sugar composition and content was performed as follows: The sugar composition (fructose, glucose, sucrose) in the fruit was analyzed by high-performance liquid chromatography (HPLC). The liquid chromatography column used was a Waters XBridge™ Amide column (250 mm × 4.6 mm, 5 μm). The mobile phase was a mixed solution of triethylamine (TEA) and acetonitrile, prepared by mixing solution A (0.2% TEA in ultrapure water) and solution B (0.2% TEA in acetonitrile) at a ratio of 24:76 (v/v). The column temperature was controlled at 30 °C. The atomization tube temperature and drift tube temperature were controlled at 60 °C, the air flow was set at 1.6 L/min, the gain value was 1.0, the flow rate was set at 1.0 mL/min, and the detection time was 18 min. To prepare the standard curve for soluble sugars, the standard was dissolved in ultrapure water, and the volume was fixed. A total of 0.0100 g (accurate to 0.0001 g) each of the L-rhamnose, D-arabinose, D-fructose, D-glucose, D-sucrose, D-lactose, and D-maltose standards was accurately weighed out. The volume of the solution was fixed at 2 mL, and the standards were mixed evenly. The concentration of the mixed standard solution was 5 ng/μL. The uniformly mixed solution was filtered by a 0.22 μm microporous membrane and stored in a refrigerator at 4 °C until use. When needed, the mixed sugar standard solution was diluted stepwise with ultrapure water to prepare 1.0 ng/μL, 0.8 ng/μL, 0.6 ng/μL, 0.4 ng/μL, 0.2 ng/μL, 0.1 ng/μL, and six other standard solutions with different concentrations, and then 10 μL samples were injected in sequence. The peak area was determined on the computer. Taking the sugar concentration as the abscissa (X) and the peak area as the ordinate (Y), a standard curve was drawn, and a linear regression equation was established **(** **Table 1** **)**.

**Table 1:** Regression equation and correlation coefficient for determination of sugar components.

Sugar Composition	Regression Equation	Related Coefficient
Rhamnose	Y=6518.60665*X^1.23616676	0.997
Arabinose	Y=5039.25102*X^1.27070354	0.997
Fructose	Y=5459.76425*X^1.25731873	0.997
Glucose	Y=5426.31563*X^1.2859865	0.997
Sucrose	Y=6190.96465*X^1.2784223	0.997
Lactose	Y=5520.03275*X^1.34197082	0.997
Maltose	Y=5753.55012*X^1.29627288	0.997

X indicates concentration, Y indicates peak area.

The extraction solution was prepared as follows: the pulp was homogenized by grinding at low temperature. Then, 1.000 g of the homogenate was accurately weighted out, 10 mL of 80% ethanol was added, and the mixture was placed in a water bath at 80 °C for 30 min. Then, the mixture was centrifuged at 4000 r/min for 10 min, the remaining residue was extracted twice with 10 mL of 80% ethanol, the filtrates were combined, and the ethanol was evaporated by decompression. The remaining solution was brought to 25 mL with ultrapure water and filtered with a 0.22 µm microporous membrane. The filtrate was used to determine the sugar composition in the jujube fruit. Under the same conditions, 1 μL of the sample was extracted. Three consecutive determinations were carried out to obtain the peak area of the sample, and the levels of soluble sugar components in the sample were calculated according to the regression equation.

### 2.3 Determination of acid content

The levels of the acid components were determined by HPLC, and the specific steps are described below. The liquid chromatography conditions were as follows: a Shimadzu Inertsil™ AQ-C18 (4.6 × 250 mm, 5 µm) from Shimadzu Experimental Equipment Co., Ltd. was used as the chromatographic column, and the mobile phase was a phosphoric acid buffer solution consisting of 0.04 mol/L K_2_HPO_4_ with a pH of 2.6~2.8 (adjusted with phosphoric acid). The instrument flow rate was set to 0.8 mL/min, and the column temperature was controlled at 30 °C. The detector was a diode array detector, and the measurement was carried out at a wavelength of 210 nm. The organic acid standard curve was prepared as follows: 0.0100 g (accurate to one tenth of a million) each of oxalic acid, tartaric acid, quinic acid, malic acid, citric acid, fumaric acid, and succinic acid standards was weighed out. The standards were dissolved in ultrapure water in a constant volume of 10 mL and mixed evenly. The concentration of this solution was 1 mg/mL. The solution was passed through a 0.22 μm microporous membrane and stored in a refrigerator at 4°C until use. When needed, the standard stock solution was dissolved in the mobile phase at an appropriate concentration to prepare standard working solutions of different concentrations. Then, 1 mL, 2 mL, 4 mL, 6 mL, 8 mL, and 10 mL of the standard stock solution were diluted stepwise to 50 mL with the mobile phase to prepare a series of mixed standard solutions with different concentrations (0.02 mg/mL, 0.04 mg/mL, 0.08 mg/mL, 0.12 mg/mL, 0.16 mg/mL, and 0.2 mg/mL), and 10 samples were injected in sequence (l0 μL injection volume). A computer was used to measure the peak area. Taking the organic acid concentration as the abscissa and the peak area as the ordinate, a standard curve was drawn, and a linear regression equation was established **(** **Table 2** **)**.

**Table 2:** Regression equation and correlation coefficient for acid component determination.

Acid Composition	Regression Equation	Related Coefficient
Oxalate	Y=17090.6091*X-37.242255	0.999
Tartaric Acid	Y=1461.77927*X+55.694918	0.998
Quinic acid	Y=478.162051*X+0.4067054	1.000
Malic Acid	Y=977.618137*X-0.0296411	1.000
Citric Acid	Y=1025.23578*X+2.4722815	0.999
Fumaric acid	Y=106776.731*X-0.9814228	0.999
Succinic acid	Y=320.213785*X-2.2578589	0.999

X indicates concentration, Y indicates peak area.

The acid extraction solution was prepared as follows: peeled pulp (1.000 g) was precisely weighed out, ground into homogenate under low temperature, extracted with 0.04 mol/L potassium dihydrogen phosphate buffer solution (pH 2.6-2.8, mobile phase), extracted with an ice bath with ultrasonication for 20 min, transferred to a 4 °C centrifuge, and centrifuged at 4000 r/min for 10 min. The supernatant was transferred to a 10 mL volumetric flask. The residue was extracted twice, dissolved to 10 mL with potassium dihydrogen phosphate buffer solution, and passed through a 0.22 µm microporous membrane for testing. The acid components in jujube fruit were quantified according to the regression equation for acid components.

The total sugar content of the fruit was measured by the anthrone sulfate method.

The total acid content of the fruit was measured by the NaOH titration method (Yan et al., 2021).

### 2.4 Statistical analysis

Analysis of the effect size indices for the two-sample t-test was performed to support the differences attested to in sugar and acid distribution in fruit research. SPSS 17 software was used for descriptive statistical analysis and origin mapping.

Heterosis was analyzed by determining the mid-parent heterosis and mid-parent heterosis rate (Bai and Ru, 2011) as follows: mid-parent heterosis (H_m_)=F_m_–V_MP_ and the mid-parent heterosis rate (R_Hm_%)=(F_m_–V_MP_)/V_MP_×100. In these equations, Fm is the mean number of phenotypic traits of the F_1_ population, and V_MP_ is the average number of phenotypic traits of both parents.

Based on the method of Gai Junyi (Gai et al., 2003), genetic analysis was conducted on 10 phenotypic data points of the F_1_ population. By calculating the maximum likelihood value (MLV) and Akaike’s information criterion (AIC) value of 11 types of genetic models of class A (polygene or a pair of major genes) and class B (two pairs of major genes), the AIC values of different models were compared (Akaike, 2003), and the suitability test was carried out to determine the optimal model. Genetic parameters such as the variance, additive effect, dominant effect, and heritability of major genes were estimated by the least square method.

The R language SEA software package was used for analysis (https://tran.rproject.org/web/packages/SEA/inde).

## 3 Results

### 3.1 Sugar and acid content of fruit

**Figure 2:**
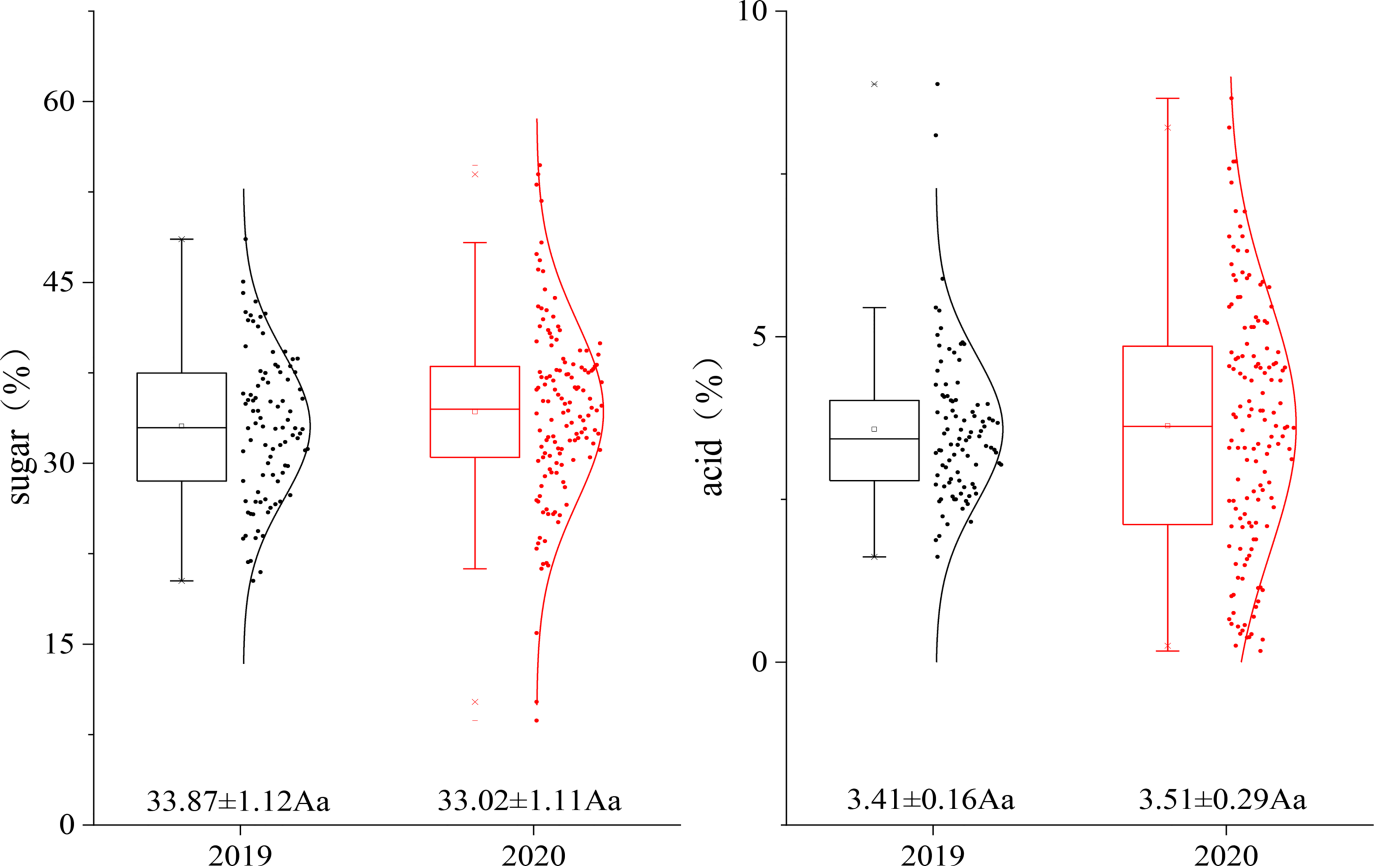
Fruit sugar–acid dispersion diagram of the F_1_ population in different years.

The sugar and acid levels of the fruit of the hybrid population were measured for two consecutive years. The average sugar levels in 2019 and 2020 were 33.87% and 33.02%, respectively, and the average acid levels in 2019 and 2020 were 3.41% and 3.51%, respectively. All the data points were basically distributed around the mean value without significant differences, and the sugar and acid levels of the fruit in the genetic population tended to be stable **(** **Figure 2** **)**.

### 3.2 Genetic tendency of sugar and acid components in fruit

**Figure 3:**
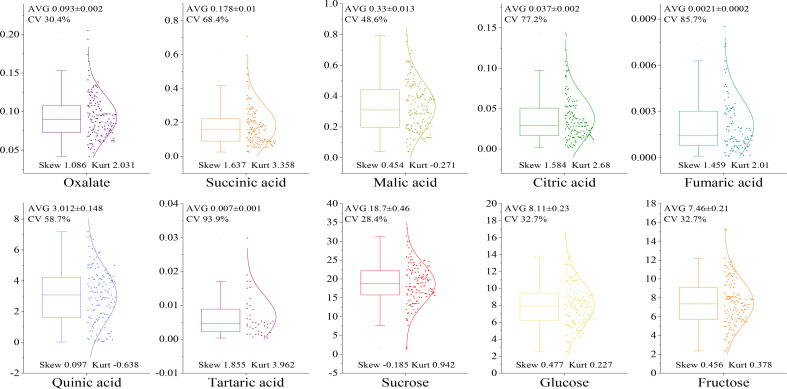
Dispersion of sugar and acid groups in fruit of the F_1_ population.

The levels of sugar and acid components in the fruit of the F_1_ hybrid population were determined as shown in **Figure 3**. The sugar components in the fruit included sucrose, glucose, and fructose. There were seven acid components: oxalic acid, malic acid, citric acid, succinic acid, tartaric acid, quinic acid, and fumaric acid. Among the acid components, the mean level of quinic acid was the highest at 3.012%, and that of tartaric acid was the lowest at 0.007%. The most abundant sugar component was sucrose at 18.70%, and the least abundant was fructose at 7.64%. The distribution range of the coefficient of variation of each component was 28.4~93.9%, and the segregation difference of the F_1_ generation was large. The distribution of malic acid and quinic acid was flatter when the kurtosis was lower than 0. When the sucrose skewness was negative (-0.185), the distribution appeared left skewed, and more data points appeared on the left side of the mean value. When the skewness of the other components was greater than 0, the distribution appeared right skewed, wherein the skewness of oxalic acid, fumaric acid, citric acid, succinic acid, and tartaric acid was greater than 1. The skewness distribution of quinic acid, malic acid, fructose, and glucose was in the range 0.097~0.477, exhibiting greater uniformity than other indicators.

**Figure 4:**
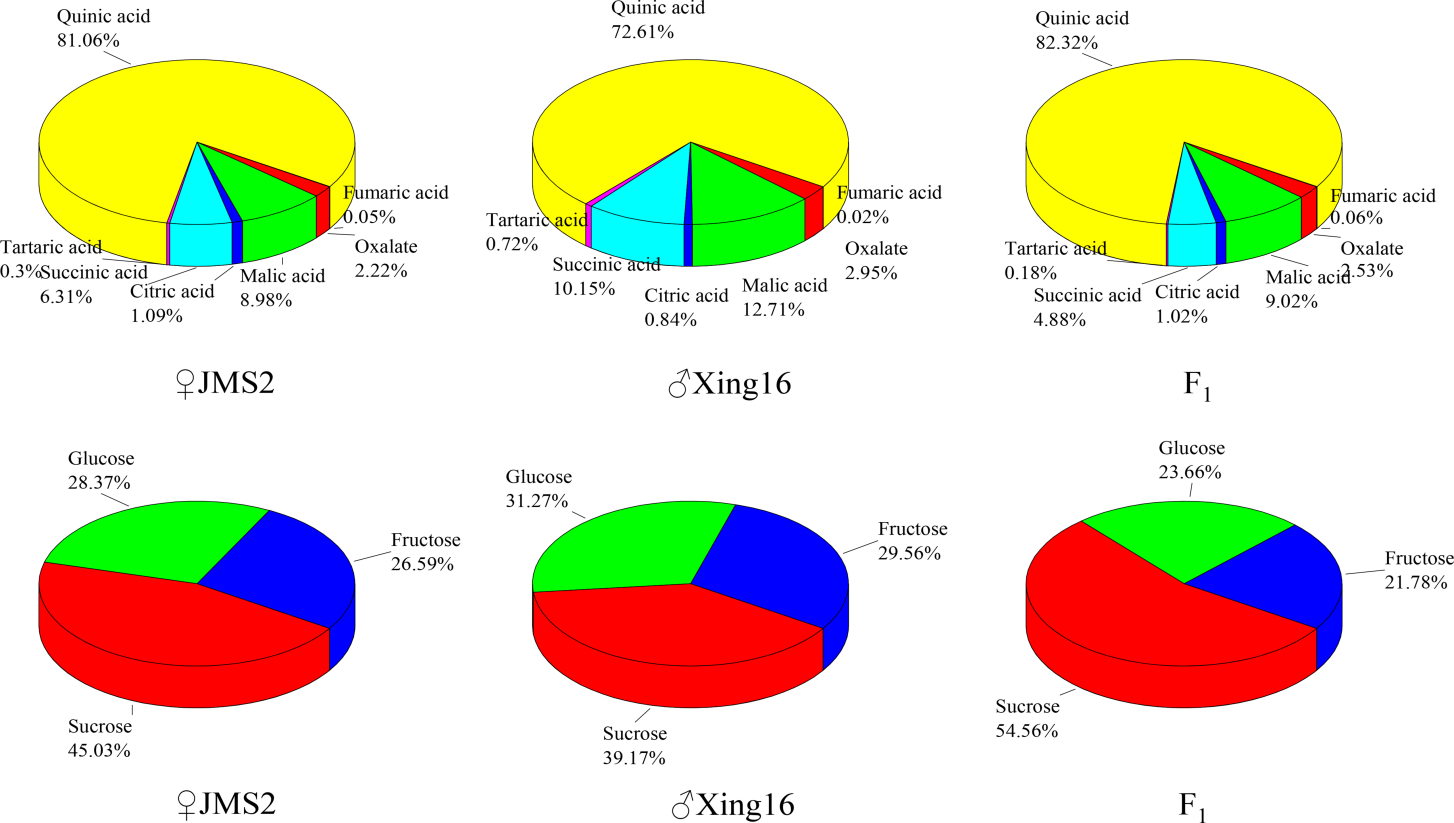
Proportion of different sugar and acid components in the parents and F_1_ population.

A comparison showed that the proportion of sugar components in the F_1_ generation and in the parents were similar **(** **Figure 4** **)**. Among them, the sugar content was the highest, with a proportion of 39.17% in the male parent and 45.03% in the female parent. The sugar composition changed in the F_1_ generation, and the average proportion of sucrose in the F_1_ generation increased to 54.56%. The fructose content of the F_1_ generation was less than that of the parents, accounting for 21.78% of the total sugar. The proportion of the acid components was similar to that of the sugars, and the acid compositions of the F_1_ generation and parents were similar, with both dominated by quinic acid. The proportion of quinic acid in the F_1_ generation was the highest at 82.32%. The abundances of other acid components tended to be similar in the female parent.

**Figure 5:**
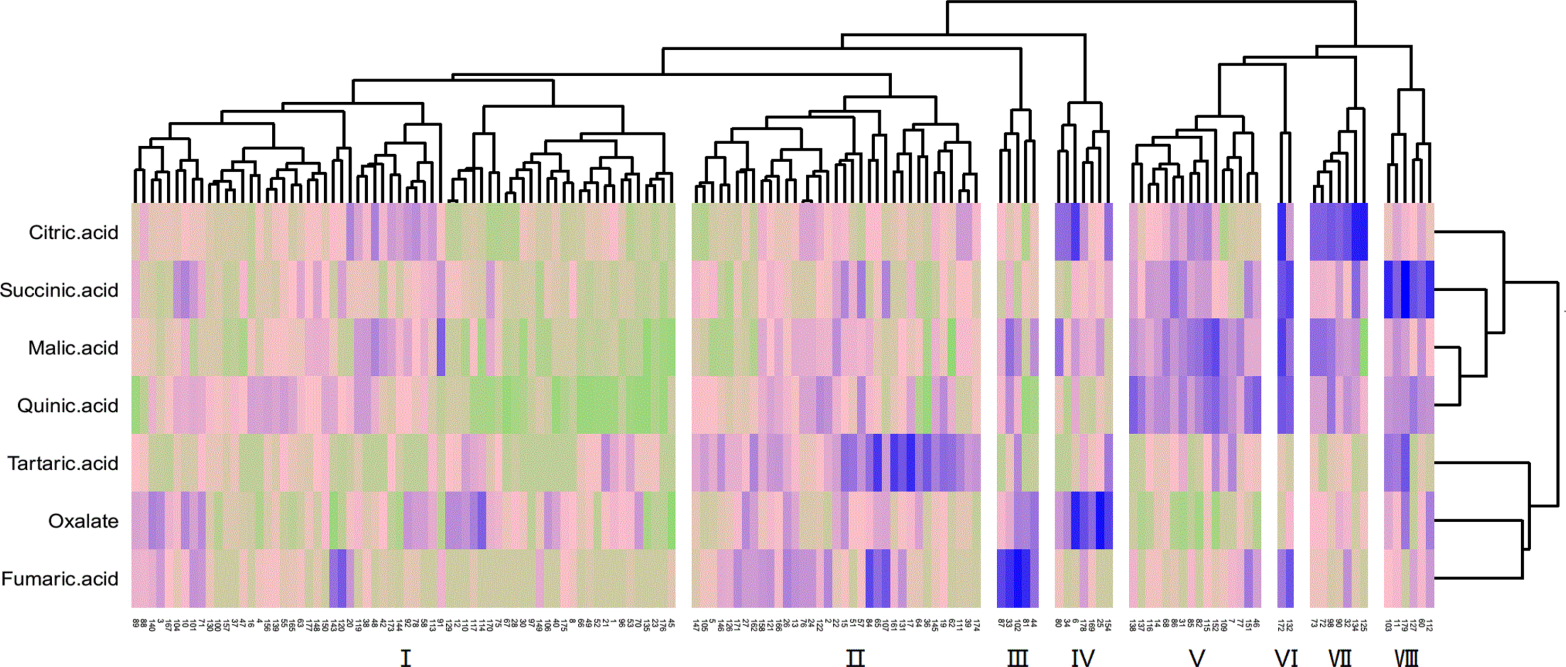
Cluster analysis of the fruit acid content of F_1_ generation jujube.

The cluster analysis of the fruit acid composition of the F_1_ hybrid population is shown in **Figure 5**. Owing to the great difference between cultivated jujube and wild jujube, the distribution of the acid components in the hybrid F_1_ generation is complex. The population could be divided into eight different acid groups with different properties. Of these, group I had the most progeny, and the abundance of the different acid components in this group was relatively average. The acid components with the highest levels in groups II, III, IV, VII, and VIII were tartaric acid, fumaric acid, oxalic acid, citric acid, and succinic acid, respectively. Group VI had only two progeny, but this group had high levels of citric acid, succinic acid, malic acid, and quinic acid, and group V had high levels of malic acid and quinic acid in the fruit.

**Figure 6:**
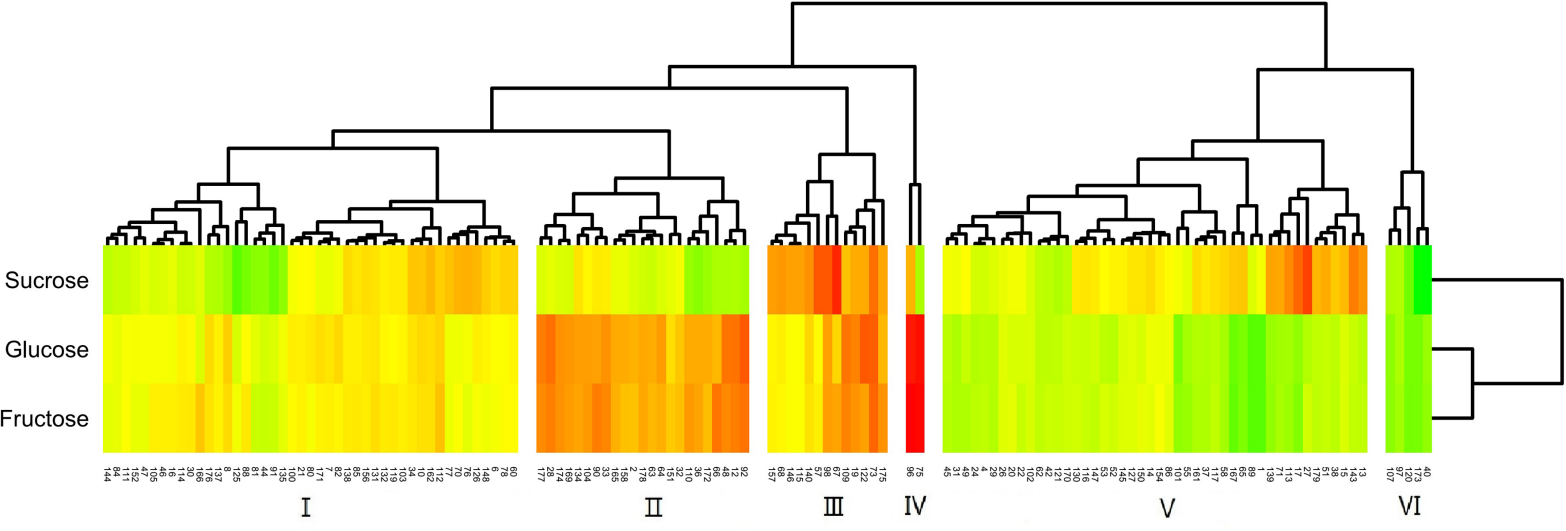
Cluster analysis of the fruit sugar content of F_1_ generation jujube.

The distribution of sugars in the fruit of the hybrids was more definite than that of acids **(** **Figure 6** **)**. The F_1_ generation was split into six groups by cluster analysis. Of these groups, group I had the most uniform sugar content of any population, and the content was also moderate. The levels of reducing sugars (glucose, fructose) in groups II and IV were high, and group IV had a higher reduced sugar content than group II. Group V was a high-sucrose group, and group III had very high levels of all three sugars. In contrast, group VI was a low-sugar group.

As shown in **Table 3**, there was no significant difference in the sugar components sucrose, glucose, and fructose between the hybrid F_1_ fruit and their parents. Significant differences were observed for succinic acid and tartaric acid among the acid components with their parents, as well as oxalic acid and malic acid. Among the sugar components, the mid-parent heterosis of sucrose was positive (5.519), and the dominant genetic effect of the genes controlling the sucrose synergism was strong, while the mid-parent heterosis of glucose and fructose was negative (-1.153, -1.250), and the genetic effect of the genes controlling this trait was strong. The average value of the three sugar components in the F1 generation exceeded the range of the parental values. Glucose and fructose had a very low parental value, while sucrose had a very high parental value.

**Table 3:** Heterosis of sugar and acid components in fruit of hybrid offspring.

Component	♀ JMS2	♂ Xing16	V_MP_	Hybrids		
				F_m_	H_m_	R_Hm_/%
Sucrose	15.003	11.357	13.180	18.699	5.519	41.88
Glucose	9.453	9.068	9.260	8.107	-1.153	-12.45
Fructose	8.859	8.570	8.715	7.465	-1.250	-14.34
Oxalate	0.100	0.191	0.146	0.093	-0.053^*^	-36.30
Malic acid	0.405	0.823	0.614	0.330	-0.284^*^	-46.25
Citric acid	0.049	0.054	0.052	0.037	-0.014	-27.71
Succinic acid	0.285	0.657	0.471	0.178	-0.293^**^	-62.12
Tartaric acid	0.014	0.047	0.030	0.007	-0.024^**^	-78.27
Quinic acid	3.658	4.701	4.180	3.012	-1.167	-27.93
Fumaric acid	0.0023	0.0012	0.0018	0.0021	0.0003	17.94

F_m_ is the average number of phenotypic traits of the F_1_ population; V_MP_ is the average value of parental phenotypic traits; H_m_ is the parent advantage = F_m_ - V_MP_; R_HM_ is the median parent dominance = (F_M_ - V_MP_)/V_MP_× 100. t-test: *p<0.05, **p<0.01.

Among the acid components, except for fumaric acid, the mid-parent heterosis was positive (0.0003), while for the other acid components, the mid-parent heterosis was negative. The genetic effect of the genes controlling the reduction in acid components was strong, with tartaric acid exhibiting the lowest mid-parent heterosis rate of -78.27%.

### 3.3 Mixed genetic analysis

The main gene+polygene mixed genetic analysis method was used to analyze the 10 fruit components of the hybrid F_1_ generation. We calculated the AIC value of the model for each of the sugar–acid components. There were some differences in the AIC values for each of the sugar–acid components, and these differences could be used to judge the degree of compatibility of each genetic model. **(** **Table 4** **)**. AIC information can balance the complexity of the estimated model with the rationality of the fitted data.

**Table 4:** AIC values from fruit polysaccharide segregation analysis of the population.

Model	Sucrose	Glucose	Fructose	Oxalate	Malic acid	Citric acid	Succinic acid	Tartaric acid	Quinic acid	Fumaric acid
0MG	830.307	644.905	622.242	-616.414	-115.259	-588.934	-194.148	-289.727	571.965	-1020.789
1MG-A	834.197	643.275	622.657	-614.409	-117.241	-631.007	-227.987	-302.188	567.616	-1047.600
1MG-AD	832.314	646.907	624.248	-621.678	-113.272	-586.930	-192.145	-287.735	567.217	-1018.786
1MG-EAD	834.197	648.905	626.242	-612.415	-111.258	-584.935	-190.150	-285.738	575.974	-1016.792
1MG-NCD	834.197	648.905	626.242	-612.415	-111.258	-584.935	-190.150	-285.738	575.974	-1016.792
2MG-A	842.694	654.920	634.165	-612.410	-113.831	-631.006	-230.181	-303.452	562.646	-1054.594
2MG-AD	838.192	647.275	626.653	-623.267	-118.443	-649.325	-240.875	-311.034	571.614	-1069.397
2MG-ADI	834.314	648.912	626.257	-626.137	-112.986	-584.930	-190.145	-285.735	565.672	-1016.787
2MG-CD	832.317	646.904	624.244	-612.415	-113.269	-586.928	-192.143	-287.734	573.949	-1018.784
2MG-EA	834.153	648.906	626.243	-614.407	-111.257	-584.935	-190.149	-285.738	575.976	-1016.791
2MG-EAD	832.151	646.906	624.243	-614.415	-113.257	-586.935	-192.149	-287.738	573.976	-1018.791

**Figure 7:**
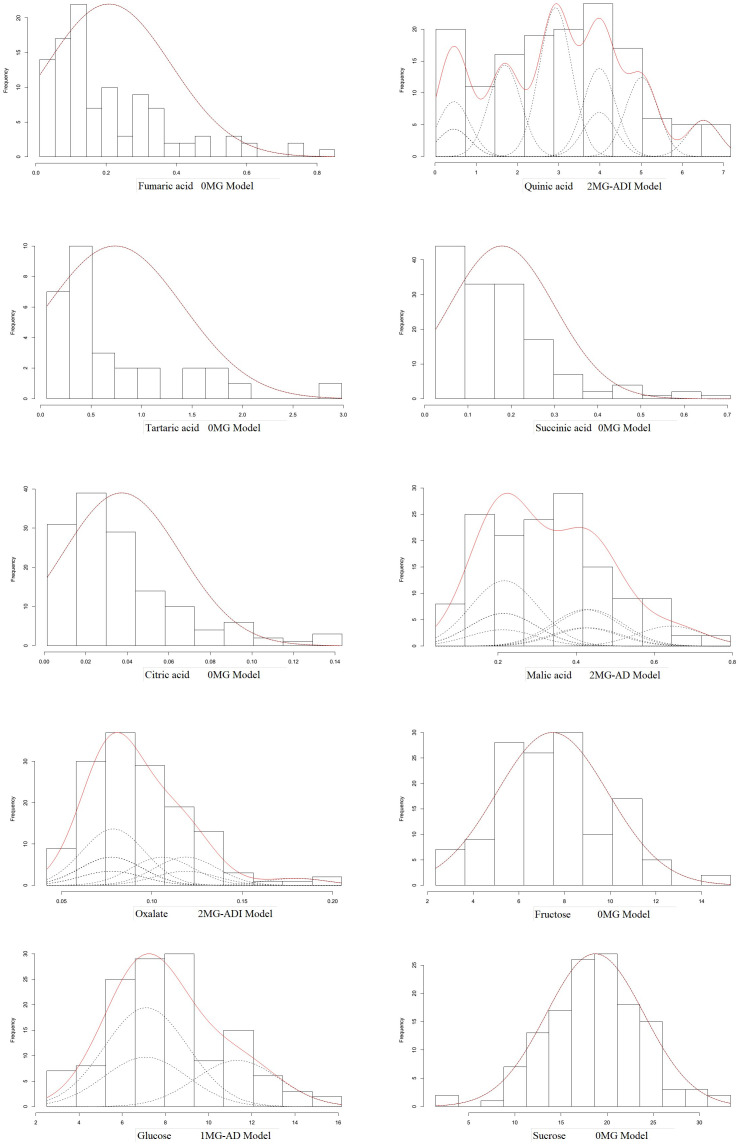
Optimal model for genetic mixture analysis of fruit sugar and acid compositions.

According to the AIC values, the minimum AIC value was determined, and the model closest to the minimum AIC value was selected as the candidate model for the uniformity test, Kolmogorov–Smirnov test, and Kolmogorov test **(** **Table 5** **)**. By comparing the calculation results of each model, the best model for different phenotypic traits was determined. Taking oxalic acid as an example, the models with low AIC values included the 2MG-ADI, 2MG-AD, 1MG-AD, and 0MG models, with values of -626.1367, -623.2667, -621.6778, and -616.4143, respectively, and these could be used as alternative models. The homogeneity test $\left({\text{U}}_{1}^{2},\;{\text{U}}_{2}^{2},\;{\text{U}}_{3}^{2}\right)$ , Smirnov test (nW^2^), and Kolmogorov test (Dn) were used to test the suitability of these four candidate models, and the model with the most significant test statistics was selected as the optimal model. The results showed that none of the values reached a significant level. Since the AIC value of 2 MG-ADI was the lowest, it was determined to be the most appropriate model. Oxalic acid is controlled by two pairs of additive-dominant-epistatic major genes. According to this rule, the optimal model for different sugar and acid components was determined (**Figure 7**).

**Table 5:** Suitability test for fruit polysaccharides.

Traits	Model	${\text{U}}_{1}^{2}$		${\text{U}}_{1}^{2}$		${\text{U}}_{3}^{2}$		nW^2^		Dn	
Sucrose	0 MG	0.0042	0.9485	0.0224	0.881	0.7206	0.396	0.0386	0.9402	0.0428	0.9578
	2 MG-EAD	0.0004	0.9842	0.0329	0.8561	0.6429	0.4227	0.0374	0.9459	0.0406	0.9736
	1 MG-A	0.0042	0.9485	0.0189	0.8908	0.639	0.4241	0.0367	0.9492	0.0421	0.9632
Glucose	1 MG-AD	0	0.9965	0.0059	0.9387	0.1056	0.7452	0.0467	0.8963	0.0452	0.9356
	0 MG	0.2514	0.6161	0.3141	0.5752	0.0899	0.7643	0.1385	0.4293	0.0846	0.2775
	2 MG-EA	0.2543	0.614	0.3033	0.5818	0.0624	0.8027	0.138	0.4309	0.0843	0.281
Fructose	0 MG	0.1821	0.6696	0.2171	0.6413	0.0445	0.8329	0.1295	0.4625	0.0885	0.2302
	1 MG-AD	0.0004	0.9849	0.0091	0.9239	0.0954	0.7574	0.0532	0.8576	0.0598	0.7011
	2 MG-EAD	0.1845	0.6675	0.2075	0.6488	0.025	0.8743	0.1294	0.4629	0.0881	0.2346
Oxalate	2 MG-ADI	0.0007	0.9786	0.0014	0.9706	0.0631	0.8017	0.0282	0.9818	0.0382	0.9794
	2 MG-AD	0.0294	0.8639	0.1943	0.6594	1.208	0.2717	0.0596	0.8187	0.0583	0.6896
	1 MG-AD	0.1233	0.7255	0.3026	0.5822	0.7069	0.4005	0.0565	0.8376	0.0575	0.7051
	0 MG	0.7795	0.3773	1.2074	0.2718	0.9524	0.3291	0.2392	0.2077	0.0996	0.1075
Malic acid	2 MG-AD	0.0049	0.944	0.0001	0.9907	0.0509	0.8216	0.0519	0.8654	0.053	0.7938
	1 MG-AD	0.02	0.8876	0.0004	0.9835	0.3973	0.5285	0.0914	0.6407	0.0675	0.5067
	0 MG	0.2418	0.6229	0.1095	0.7407	0.3376	0.5612	0.1301	0.4604	0.0819	0.2734
Citric acid	2 MG-AD	0	0.9992	0.0001	0.9929	0.001	0.9747	0.0311	0.9723	0.0439	0.941
	1 MG-AD	0.2109	0.6461	0.3526	0.5526	0.3559	0.5508	0.1267	0.4736	0.0666	0.5464
	2 MG-ADI	0.1154	0.7341	0.0748	0.7845	0.0491	0.8246	0.0681	0.7692	0.0492	0.8724
	0 MG	2.2881	0.1304	3.9036	0.0482	4.1799	0.0409	0.9372	0.0035	0.1513	0.0031
Succinic acid	2 MG-AD	0.0179	0.8937	0.0235	0.8783	0.009	0.9242	0.1108	0.5429	0.0727	0.4132
	2 MG-ADI	0.1318	0.7166	0.031	0.8602	0.4922	0.483	0.1711	0.3322	0.0976	0.1204
	1 MG-AD	0.2118	0.6454	0.3366	0.5618	0.2899	0.5903	0.1787	0.3139	0.0869	0.2142
	0 MG	2.06	0.1512	3.6145	0.0573	4.1863	0.0408	0.7823	0.0081	0.127	0.0175
Tartaric acid	2 MG-AD	0.0234	0.8784	0.0022	0.9622	0.1624	0.687	0.0497	0.8785	0.0811	0.936
	2 MG-ADI	0.0841	0.7718	0.0533	0.8175	0.0401	0.8413	0.0511	0.87	0.0868	0.8983
	1 MG-AD	0.102	0.7495	0.2917	0.5892	0.853	0.3557	0.103	0.5808	0.1249	0.5206
	0 MG	0.8067	0.3691	1.3748	0.241	1.468	0.2257	0.4732	0.0477	0.2318	0.0226
Quinic acid	2 MG-ADI	0.0007	0.9782	0	0.998	0.0092	0.9235	0.0164	0.9992	0.0351	0.9922
	2 MG-A	0	0.995	0.0001	0.9929	0.0036	0.9522	0.0321	0.9688	0.0423	0.9501
	1 MG-A	0.0004	0.984	0.0061	0.938	0.0545	0.8155	0.057	0.8342	0.0432	0.9417
	1 MG-AD	0.0115	0.9144	0.0074	0.9315	0.0052	0.9424	0.0308	0.9736	0.0431	0.943
Fumaric acid	2 MG-AD	0.0113	0.9154	0.0061	0.9379	0.0099	0.9209	0.0326	0.9668	0.0461	0.9726
	2 MG-ADI	0.0675	0.795	0.0568	0.8116	0.0028	0.9577	0.0603	0.8143	0.0598	0.8288
	1 MG-AD	0.2806	0.5963	0.3175	0.5731	0.0409	0.8397	0.2291	0.2216	0.1062	0.1778
	0 MG	1.6481	0.1992	2.4122	0.1204	1.5388	0.2148	0.7319	0.0107	0.1572	0.0103

The additive effect of the major genes of different components was positive **(** **Table 6** **)**. Among them, oxalic acid was affected by the interactions of two pairs of major genes, and the additive effects of the two pairs of genes were 0.0359 and 0.0153, while the dominant effect was negative. The effect of the first pair of genes was higher than that of the second pair of genes, and the heritability of the major gene was 68.19%. The additive and explicit components of the epistatic effect generated by the interactions of two pairs of genes were both positive. Quinic acid was also controlled positively by two pairs of major genes. The additive effect of the first major gene (2.3978) was greater than that of the second major gene (0.6313), while the dominant effect was negative. The epistatic effect of the two alleles was positive (0.6309), except that the additive effect (-0.1185) was negative. With a significant additive effect (1.7157) and a significant explicit effect (1.3984), the heritability of the major gene was 95.09%. Malic acid is controlled by two pairs of major genes. The additive effects were 0.1029 and 0.1227. The dominant effects were -0.1426 and -0.0856, respectively. Glucose was controlled by a pair of major genes. The additive effect was 2.1237, the dominant effect was -2.0943, and the heritability was 46.72%.

**Table 6:** Estimation of genetic parameters of fruit polysaccharides.

Traits	Model	*m*	*d_a_*	*d_b_*	*h_a_*	*h_b_*	*I*	*j_ab_*	*j_ba_*	*l*	*pσ^2^*	$h_{mg/\%}^{2}$
Sucrose	0MG Model											
Glucose	1MG-AD Model	9.2063	2.1237		-2.0943						3.2922	46.7237
Fructose	0MG Model											
Oxalate	2MG-ADI Model	0.1134	0.0359	0.0153	-0.0396	-0.0153	0.0152	-0.0152	0.0167	0.0202	0.0005	68.1883
Malic acid	2MG-AD Model	0.4212	0.1029	0.1227	-0.1426	-0.0856					0.0183	70.8665
Citric acid	0MG Model											
Succinic acid	0MG Model											
Tartaric acid	0MG Model											
Quinic acid	2MG-ADI Model	2.8534	2.3978	0.6313	-1.2116	-0.1188	0.6309	-0.1185	1.7157	1.3984	2.976	95.0916
Fumaric acid	0MG Model											

m, population mean square variance; d_a_, the first major-gene additive effect; d_b_, the second major-gene additive effect; h_a_, the first major-gene dominant effect; h_b_, the second major-gene dominant effect; I, additive effect; j_ab_, additive effect; j_ba_, additive effect; l, significant effect; pσ^2^, variance of major genes; $h_{mg}^{2}$ , major-gene heritability.

## 4 Discussion

Cultivated jujube evolved from wild jujube (Jin and Jun, 2003; Wang et al., 2014; Badr et al., 2020). During the evolution of a species, segregation determines whether a trait is associated with two or more genes. The experimental material is the true hybrid material obtained from hybridization and molecular identification (Jin et al., 2014). The variation coefficients of different sugars and acids in F_1_ fruit vary greatly, ranging from 28.4% to 93.9%, providing a good background for genetic analysis (Bazzaz et al., 2011). According to the analysis of the proportion of sugar and acid components in the progeny, the distribution of acid components in the F_1_ generation was similar to that of the parent, while the distribution of sugar components in the F_1_ generation was sucrose>glucose>fructose, which was the same as that of the parent, but the proportion of sucrose in the progeny was higher than that in the parent. Sucrose (54.56%) and quinic acid (82.32%) accounted for the largest proportion of sugar and acid components, with abundances in the progeny higher than those in the parents. The sucrose skewness was negative (-0.185), more data points were distributed at values higher than the mean value, and the quinic acid skewness was positive (0.097). The distribution characteristics were more uniform around the mean value. The dominant effect of the genes controlling sucrose and quinic acid showed a positive gain.

As seen in the results of the cluster analysis, the classification of acids is more complex than that of sugars. Several studies (Umer et al., 2020; Yu et al., 2022) have noted that the regulation of the acid-regulating genes in fruits is complex. In most acid groups (II, III, IV, VII, VIII), only one component stands out. The control of different acid components is independent of inheritance (Zhang et al., 2003). Through hybridization, the genes that control a certain component exhibit a unique separation in the process of gene interaction. Individuals with high levels of multiple components have a low probability of occurrence (VI groups). At the same time, there are a large number of intermediate groups (I groups), which also suggests that there is some interaction effect among the acid control genes. The distribution of reducing sugars (glucose and fructose) in the hybrid groups with different sugar components is consistent, and the probability of the occurrence of high-sugar groups in the hybrid offspring is higher.

The fumaric acid content was within the range of the parents, but the average value of the other nine components (sucrose, glucose, fructose, oxalic acid, malic acid, citric acid, succinic acid, tartaric acid, and quinic acid) for the hybrid offspring exceeded the range of the parents, and there was a phenomenon of over-parent separation. The sucrose content was higher than the range of parents. R_Hm_/% was positive (41.88%), and the other traits were lower than the range of the parents. R_Hm_/% is negative (-78.27%~-12.45%), indicating the strong dominant genetic effect of the gene controlling this trait. R_Hm_/% decreased, and according to the additive-dominant effect model (Paril et al., 2022), the mean value for the offspring was lower than the median parental value, indicating that these eight traits had low values for parent phenotypes and suggesting that the gene with reducing effects has relatively strong dominant genetic effects. The acid components showed a tendency towards the maternal direction, while the sugar components were relatively complex. Glucose and fructose showed a bias towards the male parent. The sucrose content exceeded that of the high-value parent (female parent). The selection of fine traits of a single plant in hybrid progeny has created favorable conditions, which is of great value to the breeding of hybrid jujube progeny.

The mixed major gene plus polygene inheritance model is an important model in quantitative trait research. The model was first proposed and executed in the inheritance analysis of human pedigrees (Stewart, 1969; Elston and Stewart, 1973). It was then expanded (Wang and Gai, 1998; Gai et al., 2000; Zhang et al., 2003) and widely used for the breeding of different traits in various plants (Chen et al., 2013; Xiaohong et al., 2013; Irfan et al., 2014; Lin et al., 2014; XinXin et al., 2014; Dong et al., 2019). To analyze the genetic effects of sugar and acid components in jujube fruit and improve its flavor, in this study, genetic analysis of the fruit sugar–acid composition of the cross was performed using a quantitative trait master gene + polygenic mixed genetic system (Gai et al., 2003). The analysis revealed that the heritability ( $h_{mg}^{2}$ ) of the four components (oxalate, quinic acid, malate, and glucose) ranged from 95.09 to 46.72. Genes have strong genetic effects, are not strongly affected by the environment, and can be evaluated by the identification of applied resources (Bharathi and Reddy, 2019). Glucose was controlled by one pair of additive-dominant master genes, and malate was controlled by two pairs of additive-dominant master genes. Oxalic acid and quinic acid were controlled by two pairs of additive-dominant-epistatic master genes. The additive effect of each gene was positive (0.0153~2.3978). Because additive effects can be transmitted in both the upper and lower generations, the resulting genetic effects can be additive, and the quantitative traits controlled by higher additive effects are relatively easy to achieve for breeding when lower generations are selected (Bajgain et al., 2016; Imai et al., 2019). Many studies have also indicated the strong additive effect of the sugar content of fruit (Shi et al., 2005; Liang et al., 2014; Wei et al., 2021; Wen et al., 2022). The higher mean fruit sugar content in the progeny than in the parent in the analysis of mid-parent dominance also illustrated that additive effects can be inherited in low generations. Although dominant effects were not directly heritable in the upper and lower generations, they were strongly associated with the performance of heterosis, with a negative dominant effect for the fruit indicator, which was especially strong in the progeny of the cross. Other components of progeny fruit showed a bias in the low-affinity direction (Hake and Rossibarra, 2015; Zolkin et al., 2021). The phenomenon of ultralow affinity was also observed. In addition, the best model for fumaric acid, succinic acid, tartaric acid, citric acid, sucrose, and fructose was the A-0 model without master gene control, indicating that these component distributions could be attributed to the additive effects of multiple minor-effect genes. More emphasis should be placed on the utilization of additive effects in the breeding of hybrid jujube. It is feasible to apply the single segregating generation F_1_ segregant analysis method for the mixed genetic model to study the genetic effect in the jujube population. The detection of major genes should be combined with the results of molecular marker studies (Liu et al., 2014) and quantitative trait locus (QTL) validation analysis in jujube, which may provide a theoretical basis for molecular marker-assisted selection in this plant.

## Data availability statement

The original contributions presented in the study are included in the article/supplementary material. Further inquiries can be directed to the corresponding authors.

## Author contributions

YZ, WZ, YF, WC, WJ, LMe, and LMi planned the research. YX and DM performed experiments. YZ and DM analyzed data. YZ and ZC tested the results. YZ and LMi wrote the paper. All authors contributed to the article and approved the submitted version.
